# Changes in the menstrual hygiene management facilities and usage among Bangladeshi school girls and its effect on school absenteeism from 2014 to 2018

**DOI:** 10.1080/16549716.2023.2297512

**Published:** 2024-01-17

**Authors:** Farjana Jahan, Noshin Sayiara Shuchi, Abul Kasham Shoab, Mahbub-Ul Alam, Sk. Md. Kamrul Bashar, Khairul Islam, Hasin Jahan, Mahadi Hasan, Md. Masud Alam, Mahbubur Rahman

**Affiliations:** a Environmental Health and WASH, International Centre for Diarrheal Diseases Research, Bangladesh (icddr,b), Dhaka, Bangladesh; b School of Civil Engineering, University of Leeds, Leeds, UK; c WaterAid, South Asia Region Bangladesh, Dhaka, Bangladesh; d Demography and Health Wing, Bangladesh Bureau of Statistics, Dhaka, Bangladesh

**Keywords:** Menstrual hygiene management, school WASH, adolescent girls, school absenteeism, menstrual hygiene facilities

## Abstract

**Background:**

The lack of menstrual hygiene management (MHM) information and facilities in schools is a major contributor to adolescent girls’ school absenteeism in low- and middle-income countries like Bangladesh.

**Objectives:**

This paper examines the changes over time in school MHM facilities, knowledge and perceptions among adolescent girls, in relation to school absenteeism between 2014 and 2018 in Bangladesh.

**Methods:**

We examined changes in MHM and school absenteeism among schoolgirls using nationally representative data from the Bangladesh National Hygiene Baseline Survey 2014 and National Hygiene Survey 2018. Given the repetitive nature of our data and its clustering within participants, our method included performing descriptive analysis, bivariate analysis, and multivariate Generalised Estimating Equation (GEE) modelling to analyse these changes.

**Results:**

Results showed that adolescent girls’ menstruation-related absenteeism decreased between 2014 and 2018. Percentage of adolescents who missed school decreased from 25% to 14% (PD: −11; CI: −16 to −6.1), while the average number of missed days reduced from 2.8 to 2.5 (PD: −0.33; CI: −0.57 to −0.10). In the GEE model, we found that living in rural areas (coef: −5.6; CI: −10.06 to −1.14), parental restrictions on going outside (coef: 4.47; CI: 0.75 to 8.2), education levels of girls (coef: −9.48; CI: −14.17 to −4.79), girl’s belief that menstruation affects school performance (coef: 23.32; CI: 19.71 to 26.93), and using old cloths (coef: −4.2; CI: −7.6 to −0.79) were significantly associated with higher absenteeism. However, participant’s age, type of school, knowledge of menstruation before menarche, receiving information regarding MHM, separate place for changing absorbents, and separate latrine and urine facility were not significantly associated with the changes in absenteeism over time.

**Conclusion:**

This paper emphasised the associations between changes in school absenteeism, parental restrictions on students, students’ education levels, and menstruation-related misperceptions. Ongoing research, policy reviews, and targeted interventions to improve MHM perceptions among girls are required to provide long-term benefits for adolescent girls in Bangladesh.

## Background

Menstrual hygiene management (MHM) in schools is subpar due to limited access to menstrual hygiene knowledge and facilities [1,2]. Adolescents must manage their menstruation at home and at school, as it is a vital part of their lives [3–5]. Worldwide studies conducted over the last ten years have demonstrated the difficulties schoolgirls encounter in managing their menstruation [6]. These challenges include limited pre-menstrual information, poor education, lack of social support from families, peers, and teachers, and restricted access to hygienic facilities [7]. These difficulties lead to gender discrimination in schools and pervasive menstruation-related stigma, causing emotional discomfort and impeding girls’ education [8–10].

In order to meet their menstrual needs at school, young teenage girls from impoverished backgrounds must overcome a variety of economic, social, and cultural obstacles around the globe, which are most pronounced in low- and middle-income countries [4,9,10]. Adolescents in Bangladesh, like those in other developing countries, lack access to adequate MHM information and facilities at home and at school [4,6,10]. As a result, as adolescent girls reach menarche, absenteeism rises [7,8].

Adolescents are frequently misled and unprepared for menstruation due to a lack of knowledge and MHM facilities and practices and social stigma [11,12]. Since they spend six hours in school, it’s critical that they have access to gender-specific sanitation facilities that are both clean and functional [3]. Poor menstrual hygiene habits are frequently caused by inadequate water, sanitation, and hygiene (WASH) facilities, a lack of privacy, and a lack of gender-specific wash facilities at schools [3,8,10]. According to two studies conducted in Bangladesh, only 9% of schools have access to soap, water, and disposal bins in the toilets, and 82% of enrolled adolescents find their schools’ facilities inappropriate for managing their periods [8,13,14]. Another study in rural Bangladesh indicated that just around 35% of adolescents were aware of menstruation, and 64% had a negative reaction to it, including reported shock, panic, confusion, tension, fear, shame, or embarrassment at menarche [15]. These elements can have long-term effects and lead to absenteeism, subpar academic performance, and reduced educational achievement for females [16–18]. This is a complex issue brought on by a lack of appropriate education and a sanitary environment [19,20].

As the number of schoolgirls entering adolescence age is increasing, emphasising proper menstrual management in schools is essential [14,15]. The National Hygiene Survey 2014 found that Bangladeshi school-going adolescents missed an average of 2.8 days per period [19]. Adolescents in Bangladesh adopt various menstrual management practices depending on their customs and available resources, not all of which are healthy [21]. In a study conducted in rural Bangladesh, it showed that more than 51% of adolescents lacked sufficient knowledge about menstruation, and many of them felt uncomfortable discussing it [21]. Adolescent girls who practice poor menstrual hygiene may develop cervical cancer, urinary tract infections, reproductive tract infections (RTI), pregnancy problems, and abdominal pain [22–24]. There is a large gap between rural and urban areas of Bangladesh when it comes to the availability of sanitation facilities for school-aged adolescents [25].

Bangladesh’s educational environment changed significantly between the hygiene surveys conducted in 2014 and 2018 [12,26,27]. To promote a positive learning environment, the Ministry of Education selected female teachers and mandated upgraded facilities for MHM education in 2015 [28]. MHM was integrated into the National Curriculum in 2016 with the collaborative efforts of national and international organisations. The Water and Sanitation Sector Development Plan (2011–25) of the government highlighted gender-appropriate WASH facilities and MHM guidelines, demonstrating a commitment to menstrual hygiene in schools [29].

Students have expressed their concern with the lack of proper sanitary facilities and understanding regarding MHM, as well as specific recommendations [20]. Recognising the changing nature of these challenges and the possible impact of interventions, it is critical to analyse changes over time to determine the effectiveness of efforts targeted at improving MHM and addressing school absenteeism. However, there are few research that compare changes in MHM facilities and school student absenteeism over time. Therefore, the purpose of this research is to gain a better understanding of the changes in menstrual hygiene knowledge, management, school facilities, and school absenteeism from 2014 to 2018 among national, urban, and rural areas and to propose possible initiatives to improve the situation.

## Methods

### Data sources

In this section, the study drew on data from two national-level surveys: The Bangladesh National Hygiene Baseline Survey 2014 and the National Hygiene Survey 2018. These national-level surveys investigated a variety of areas, including water, sanitation, hygiene, and knowledge. The analysis was conducted using a sizeable sample size. The surveys were divided into four main categories: household-level hygiene component, school hygiene including MHM, food hygiene in restaurants and among street food vendors, and health facility hygiene [30,31].

### Data sampling procedure

This section contains a full description of the data sampling process used in this study. A nationally representative sample was selected for 2014 and was split into rural and urban areas. A two-stage cluster sampling procedure was used to identify the schools. The study team divided Bangladesh into two strata, rural and urban, from where 86,925 rural villages and 10,000 urban mahallas (locality) were selected as representative population clusters. The study team used probability proportional to size (PPS) sampling to select a total of 100 clusters: 50 for rural and 50 for urban areas. Based on the indicator ‘schools having soap and water at handwashing location’ from the rural school survey, a sample size of 2333 girls were determined, with 10% discrepancy between rural and urban schools and the design effect of 12.0, power of 0.8, and α of 0.05 to get the sample size [30].

**Figure 1.**
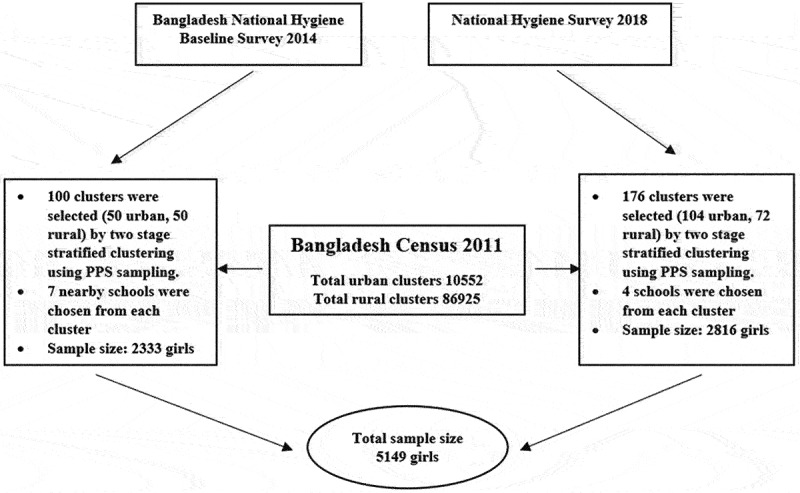
Flowchart: study sampling procedure.

A total of 176 Enumeration Areas (EAs) were selected in 2018 utilising PPS sampling from the 293,570 EAs in Bangladesh. These EAs organised into 176 clusters, of which 104 were urban and 72 were rural. The sample framework, which was modified for rural areas, was guided by the 2011 Bangladesh Population and Housing Census. Field teams identified primary and secondary schools close to each cluster’s homes, choosing four primary and six secondary schools out of every ten. A sample of 704 schools were taken from the 176 clusters. Using data from the baseline study, a sample size of 2816 girls (16 per cluster) was determined, with a precision of 4.0% and a design effect of 2.5 [31]. For this study, the sample size was 5149 girls. The data sample procedure is reported in Figure 1.

### Study site and study population

This section describes the study sites and participant selection criteria for the Bangladesh National Hygiene Baseline Survey 2014 and the National Hygiene Survey 2018. The baseline data in 2014 were collected from seven nearby schools from each of the 100 clusters. The schools were eligible if those were government or registered primary or high school and if the headmaster or class teacher gave consent along with the students’ assent. The participants of this study were four randomly selected girls from each school, who had menstrual experience and enrolled in grades 2 to 9.

For the 2018 survey, study sites comprised four nearby schools from all households of the 176 clusters. From each of the schools, four girls with menstruation experience from class V were selected for primary schools and for secondary schools, and girls were selected from classes VI–X. Similar to the baseline survey, four girls who menstruated before the survey were selected for the interview by the headmasters at each school [31].

Eligibility criteria remained consistent for both the Bangladesh National Hygiene Baseline Survey 2014 and the National Hygiene Survey 2018, government or registered primary or high school and headmaster gave informed consent; class teacher gave consent and students gave assent.

### Data collection

Primary data for the Bangladesh National Hygiene Baseline Survey 2014 were collected by icddr,b, and included participant-reported and spot-check indicators for school handwashing. Data include information on handwashing, water, sanitation, and hygiene practices. Four chosen students were interviewed using a standardised questionnaire by trained female data collectors, who also performed spot checks for MHM facilities. The National Hygiene Survey 2018 used in-person interviews, spot checks, and handwashing demonstrations by Bangladesh Bureau of Statistics (BBS) field experts to gather primary data. 2018’s data gathering took place between March and May.

### Study measures

Outcome variable of this study was absenteeism from school due to menstruation. School absenteeism was defined having missed from school during their menstruation based on participant’s responses.

In the study, we considered changes over time from 2014 to 2018 as an exposure variable. To investigate the changes over the two-time tenure, we appended the two datasets and created a composite ‘time’ variable, where the time point of 2014 was considered as 0 and 2018 was 1.

In our study, we considered some factors as independent variables, which were age, place of residence, type of school (primary and secondary schools), participants level of education (primary and secondary level), knowledge of menstruation before menarche, believe menstruation interferes school performance, participants received any information regarding MHM from school, parents restriction in attaining school during menstruation, using old cloth, separate place for changing MHM materials, and separate latrine and urine facilities [30,31].

### Statistical analyses

To comprehensively address our research objectives, we employed diverse analytical approaches, including descriptive analysis, bivariate analysis, and multivariate Generalised Estimating Equation (GEE) modelling.

We reported frequencies and percentages for the categorical variables. We reported means for continuous variables since those were normally distributed. The reported percentages and means are weighted national estimates to adjust for rural/urban balance, which we calculated using a sampling weight.

We employed weighting variables during the analysis to account for the complex sampling design and ensure the representativeness of the survey. This process was required to produce accurate statistical findings that accurately reflected the target population. Since the total number of clusters was different for urban and rural areas, to adjust this clustering effect, we multiplied the urban and rural data with weight factors: Rural – 50/86,925, Urban – 50/105,52 for 2014 and Rural – 104/86,925, Urban – 72/10,552 for 2018 [30,31].

We appended the data from 2014 to 2018, which allowed us to construct a comprehensive dataset that covered a large amount of time and revealed potential changes in MHM-related school absences from 2014 to 2018. Rigorous procedures were utilised to clean, document, and assess the data that were collected in accordance with the suggestions made in the survey report.

We performed descriptive analysis to provide an estimation of the study’s participants’ primary characteristics and changes in school absenteeism. Additionally, a bivariate analysis (chi-square test) was conducted to investigate the unadjusted associations between time changes and absenteeism from school. Moreover, we performed the multivariate GEE models to evaluate the adjusted associations between time changes and school absenteeism. We used STATA 15 software version to analyse the data.

## Results

In this paper we presented the changes in MHM-related knowledge and perception among schoolgirls, the MHM-related practices, MHM facilities and education in schools, and MHM-related school absenteeism. We compared the changes both school wise (urban and rural) and overall.

### Demographic characteristics of the respondents

Participants’ demographic characteristics shifted dramatically between 2014 and 2018 (Table 1). In 2018, rural schools had a higher proportion of girls than urban schools (59% vs 47% rural in 2014). More participants (61% vs 39% in 2018) were enrolled in secondary school rather than in primary school and had a secondary education level (65% vs 35% in 2018). The mean age of the interviewed girls was 12.5 to 13.2 years, respectively, from 2014 to 2018.

**Table 1.** Demographic characteristics of the study participants.

	2014 (*N* = 2333)		2018 (*N* = 2816)			
Indicators	Rural schools (n, %*)	Urban schools (n, %*)	All schools (n, %*)	Rural schools (n, %*)	Urban schools (n, %*)	All schools (n, %*)
Place of residence	1106 (47)	1227 (53)	2333	1664 (59)	1152 (41)	2816
Type of school						
Primary school	1005 (91)	1139 (93)	2144 (91)	644 (39)	480 (42)	1124 (39)
Secondary school	101 (9)	88 (7)	189 (9)	1020 (61)	672 (58)	1692 (61)
Education level of participants						
Primary level	780 (71)	794 (65)	1574 (70)	572 (35)	436 (38)	1008 (35)
Secondary level	326 (29)	433 (35)	759 (30)	1083 (65)	715 (62)	1798 (65)
Age of the participants						
9–12	593 (54)	625 (51)	1218 (53)	544 (33)	403 (35)	947 (33)
13–17	513 (46)	601 (49)	1114 (47)	1107 (67)	746 (65)	1853 (67)
Mean age of students	12.5	12.5	12.5	13.2	13.2	13.2
Mean age at first menstruation (years)	11.5	11.5	11.5	11.7	11.6	11.7

*Weighted percentage for rural/urban balance and school size.

### MHM-related knowledge and perception among schoolgirls

Awareness of menstruation before menarche has increased significantly, growing from 36% in 2014 to 53% in 2018. However, in 2018, more girls reported that they believe menstruation interfered with their school performances (PD: 13; CI: 7.5, 19). Also, knowledge of the implications of inadequate MHM decreased from 57% in 2014 to 41% in 2018. The perception regarding menstruation did not change significantly from 2014 to 2018 (Table 2).

**Table 2.** Changes in the knowledge and perception of adolescent schoolgirls in urban and rural schools of Bangladesh from 2014 to 2018.

Indicators	Rural school (*N* = 2757)		PD (CI)	Urban school (*N* = 2375)		PD (CI)	All school (*N* = 5132)		PD (CI)
	2014 (n, %*)	2018 (n, %*)		2014 (n, %*)	2018 (n, %*)		2014 (n, %*)	2018 (n, %*)	
**Knowledge on menstruation**†	***N* = 1106**	***N* = 1651**		***N* = 1226**	***N* = 1149**		***N* = 2332**	***N* = 2800**	
Knew about menstruation before menarche	395 (36)	882 (53)	17 (12, 23)	467 (38)	613 (53)	15 (10, 20)	862 (36)	1495 (53)	17 (12, 22)
Believes menstrual problems interfere with school performance	345 (31)	757 (46)	14 (7.9, 20)	411 (34)	457 (40)	5.3 (−1.5, 12)	756 (31)	1214 (45)	13 (7.5, 19)
Believes menstruation is a female/personal matter	528 (48)	704 (43)	−7.3 (−16, 1.7)	620 (51)	463 (40)	−10 (−18, -2.0)	1148 (48)	1167 (42)	−7.6 (−16, 0.47)
Knows about the implication of inadequate management of menstrual hygiene	625 (57)	677 (41)	−15 (−25, -6.1)	732 (60)	523 (46)	−15 (−24, -6.2)	1357 (57)	1200 (41)	−15 (−24,−7.0)
**Perception regarding menstruation**									
A normal biological process for women	433 (39)	683 (41)	2.3 (−6.0, 11)	526 (43)	555 (48)	4.8 (−3.7, 13)	959 (40)	1238 (42)	2.6 (−4.9, 10)
An illness of females	117 (11)	76 (5)	−6.8 (−12, −1.5)	112 (9)	69 (6)	−3.2 (−7.0, 0.72)	229 (10)	145 (5)	−6.3 (−11, -1.7)
Curse of God	6 (1)	36 (2)	1.7 (.22, 3.2)	6 (1)	27 (2)	2.0 (.30, 3.6)	12 (1)	63 (2)	1.7 (0.40, 3.1)

PD = Proportion difference; CI = Confidence interval.

Positive (Yes) responses from students were considered.

*Weighted percentage for rural/urban balance and school size.

^†^Multiple answers under this variable.

### Changes in MHM-related practices among schoolgirls

The use of sanitary pads increased dramatically, going from 10% in 2014 to 57% in 2018. The reuse of cloth or rags during menstruation, on the other hand, significantly decreased, falling from 82% in 2014 to 38% in 2018. There was a decline in disposing menstrual absorbents in open dumping place, and there was an increase in the use of toilet pans for disposal (Table 3).

**Table 3.** Changes in practices among adolescent schoolgirls in urban and rural schools of Bangladesh from 2014 to 2018.

Indicators	Rural school (*N* = 2757)		PD (CI)	Urban school (*N* = 2375)		PD (CI)	All school (*N* = 5132)		PD (CI)
	2014 (n, %*)	2018 (n, %*)		2014 (n, %*)	2018 (n, %*)		2014 (n, %*)	2018 (n, %*)	
** Practices regarding menstrual hygiene**									
** Materials used during menstruation**	***N* = 1106**	***N* = 1651**		***N* = 1226**	***N* = 1149**		***N* = 2332**	***N* = 2800**	
Reused cloth/rag	922 (83)	654 (40)	−37 (−40, −34)	887 (72)	311 (27)	−39 (−43, −35)	1809 (82)	965 (38)	−37 (−40, −34)
Sanitary pad	97 (9)	915 (55)	39 (36, 41)	258 (21)	781 (68)	39 (36, 42)	355 (10)	1696 (57)	38 (36, 41)
Others (new cloth/cotton/tissue/jute of garments/nothing)	87 (8)	82 (5)	−2.5 (−6.3, 1.4)	81 (7)	57 (5)	−1.8 (−5.0, 1.3)	168 (8)	139 (5)	−2.4 (−5.9, 1.0)
** Materials used for cleaning the cloth**	***N* = 962**	***N* = 723**		***N* = 943**	***N* = 355**		***N* = 1905**	***N* = 1078**	
Soap/detergent and water	864 (90)	651 (90)	0.33 (−2.9, 3.5)	814 (86)	318 (90)	3.5 (−1.5, 8.6)	1678 (89)	969 (90)	0.68 (−2.3, 3.7)
Soap, disinfectant with water	16 (2)	13 (2)	0.13 (−1.1, 1.4)	35 (4)	9 (3)	−1.4 (−4.3, 1.6)	51 (2)	22 (2)	−.04 (−1.3, 1.2)
Water	53 (5)	20 (3)	−3.3 (−6.5, −.16)	838 (4)	8 (2)	−2.1 (−5.6, 1.4)	91 (5)	28 (3)	−3.2 (−6.0, −.31)
Didn’t clean	25 (3)	29 (4.0)	1.3 (−.34, 2.9)	44 (5)	14 (4)	−.76 (−4.0, 2.5)	69 (3)	43 (4)	1.1 (−0.44, 2.6)
** Washed reused cloth with soap, water and dried in sunlight**	276 (29)	177 (25)	−3.1 (−9.6, 3.3)	242 (26)	76 (22)	−2.8 (−10, 4.5)	518 (28)	253 (24)	−3.1 (−8.9, 2.8)
	***N* = 937**	***N* = 694**		***N* = 899**	***N* = 342**		***N* = 1836**	***N *= 1036**	
** Stored menstrual cloth normally like other clothes**	56 (6)	65 (9)	3.5 (.67, 6.3)	75 (8)	56 (16)	7.0 (2.8, 11)	131 (6)	121 (10)	3.8 (1.2, 6.3)
** Dried menstrual cloth outside the house and in sunlight**	295 (31)	193 (28)	−2.8 (−9.7, 4.0)	268 (30)	84 (25)	−3.8 (−12, 4.1)	563 (31)	277 (28)	−2.9 (−9.1, 3.3)
	***N* = 1055**	***N *= 1651**		***N* = 1162**	***N* = 1149**		***N* = 2217**	***N* = 2800**	
** Disposal of menstrual absorbents if there’s no separate place**†									
Openly disposed	38 (4)	28 (2)	−2.1 (−4.2, .06)	33 (3)	18 (2)	−1.3 (−3.7, 0.95)	71 (4)	46 (2)	−2.0 (−3.9, −0.09)
Disposed inside toilet pan	55 (5)	375 (23)	17 (13, 22)	30 (3)	239 (21)	19 (13, 24)	85 (5)	614 (22)	17 (13, 21)
Didn’t change at school	897 (85)	1220 (74)	−11 (−16, −4.7)	1038 (89)	8701 (76)	−13 (−19, −7.3)	1935 (86)	2,090 (74)	−11 (−16, −5.6)

PD = Proportion difference; CI = Confidence interval.

Positive (Yes) responses from students were considered.

*Weighted percentage for rural/urban balance and school size.

^†^Multiple answers under this variable.

### Changes in MHM facilities

#### MHM facilities

The overall proportion of schools with separate facilities for changing menstruation materials ascended from 3% in 2014 to 8% in 2018 (PD: 4.8; CI: 2.0–7.5). With an increase from 4% in 2014 to 23% in 2018 (PD: 19; CI: 15–24), there was a significant improvement in the availability of a dustbin for disposing of menstrual products in girls’ toilets (Table 4).

**Table 4.** Changes in menstrual hygiene management facilities and education in urban and rural schools in Bangladesh from 2014 to 2018.

Indicators	Rural schools (*N* = 2757)		PD (CI)	Urban schools (*N* = 2375)		PD (CI)	All schools (*N* = 5132)		PD (CI)
	2014 (n, %*)	2018 (n, %*)		2014 (n, %*)	2018 (n, %*)		2014 (n, %*)	2018 (n, %*)	
	***N* = 1106**	***N* = 1651**		***N* = 1226**	***N* = 1149**		***N* = 2332**	***N* = 2800**	
Separate facility at school for changing menstrual materials	38 (3)	137 (8)	4.6 (1.6, 7.6)	29 (2)	98 (8)	6.1 (2.5, 9.7)	67 (3)	235 (8)	4.8 (2.0, 7.5)
	***N* = 38**	***N* = 137**		***N* = 29**	***N* = 98**		**N = 67**	***N* = 235**	
The separate facility at school is safe, clean, and private to use	25 (66)	111 (81)	11 (-10, 33)	21 (72)	87 (89)	13 (−4.3, 30)	46 (66)	198 (82)	12 (−7.7, 31)
The separate facility at school is used by students	20 (53)	95 (69)	16 (-6.4, 39)	13 (45)	72 (73)	26 (4.6, 48)	33 (52)	167 (70)	17 (-3.4, 38)
	***N* = 1106**	***N* = 1651**		***N* = 1226**	***N* = 1149**		***N* = 2332**	***N* = 2800**	
Dustbin for throwing menstrual materials in girls’ toilet	37 (3)	368 (22)	19 (14, 24)	63 (5)	312 (27)	20 (15, 27)	100 (4)	680 (23)	19 (15, 24)
Availability of hygiene kit (Dettol, cotton/rag, soap) during menstruation	11 (1)	160 (10)	9.5 (4.7, 14)	12 (1)	205 (18)	20 (14, 26)	23 (1)	365 (11)	11 (6.1, 15)
Distribution of menstruation materials at school	8 (1)	98 (6)	5.9 (2.4, 9.5)	13 (1)	116 (10)	9.9 (5.8, 14)	21 (1)	214 (6)	6.4 (3.3, 9.6)
**Menstrual hygiene management education in schools**									
Received any information regarding MHM before menarche from school	22 (2)	366 (22)	22 (17, 26)	95 (8)	304 (26)	18 (13, 22)	117 (3)	670 (23)	21 (17, 25)
Counselling for students regarding MHM	60 (5)	323 (20)	14 (9.4, 18)	172 (14)	279 (24)	10 (4.7, 16)	232 (6)	602 (20)	13 (9.5, 17)
Separate classes held for girls	9 (1)	67 (4)	3.4 (1.2, 5.5)	11 (1)	76 (7)	6.2 (2.8, 9.5)	20 (1)	143 (4)	3.7 (1.7, 5.6)

PD = Proportion difference; CI = Confidence interval.

Positive (Yes) responses from students were considered.

*Weighted percentage for rural/urban balance and school size.

#### MHM education in schools

The provision of MHM education in school curriculum and classes has significantly improved in all schools overall. The distribution shows, from 3% in 2014 to 23% in 2018 (PD: 21; CI: 17–25), more girls were informed by their schools about MHM before menarche. Additionally, there was an improvement in the percentage of students who received menstrual hygiene counselling, which increased from 6% in 2014 to 20% in 2018 (PD: 13; CI: 9.5–17) (Table 4).

### Changes in school absenteeism due to menstruation

The findings in Table 5 provide an extensive overview of the changes in school absenteeism caused by menstruation and associated factors among adolescents attending urban and rural schools in Bangladesh from 2014 to 2018.

**Table 5.** Changes in school absenteeism due to menstruation in urban and rural schools in Bangladesh from 2014 to 2018.

Indicators	Rural schools (*N* = 2757)		PD (CI)	Urban schools (*N* = 2375)		PD (CI)	All schools (*N* = 5132)		PD (CI)
	2014 (n, %*)	2018 (n, %*)		2014 (n, %*)	2018 (n, %*)		2014 (n, %*)	2018 (n, %*)	
	***N* = 1106**	***N* = 1651**		***N* = 1226**	***N* = 1149**		***N* = 2332**	***N* = 2800**	
Missed schools during menstruation	287 (26)	246 (15)	−11 (−17, −5.6)	247 (20)	117 (10)	-11 (−16, −5.2)	534 (25)	363 (14)	−11 (−16, −6.1)
Mean number of missed days at school due to menstruation	2.8	2.5	−.36 (−.61, −.10)	2.6	2.6	−.12 (−.45, .19)	2.8	2.5	−.33 (−.57, −.10)
Missed classes at school due to menstruation	449 (41)	515 (31)	−10 (−16, −3.3)	482 (39)	249 (22)	−18 (−24, −12)	931 (40)	764 (30)	−10 (−17, −4.9)
**Reasons for missing schools during menstruation**†	***N* = 287**	***N* = 246**		***N* = 247**	***N* = 117**		***N* = 534**	***N* = 363**	
No separate place to change rag/cloths	22 (8)	35 (14)	5.8 (.77, 11)	26 (11)	20 (17)	4.8 (−2.9, 12)	48 (8)	55 (14)	5.7 (1.1, 10)
Felt uncomfortable	149 (52)	128 (52)	−1.7 (−13, 9.2)	138(56)	60 (51)	−5.4 (−18, 6.9)	287 (52)	188 (52)	−2.04 (−12, 7.9)
No water/soap	6 (2)	10 (4)	1.8 (−1, 4.7)	3 (1)	6 (5)	3.1 (.18,6.1)	9 (2)	16 (4)	2.0 (−.62,4.5)
	***N* = 1106**	***N* = 1651**		***N* = 1226**	***N* = 1149**		***N* = 2332**	***N* = 2800**	
Parent’s restriction to go out during menstruation	282 (26)	235 (14)	−12 (−18, −5.6)	322 (26)	101 (9)	−19 (−26, −12)	604 (26)	336 (14)	−13 (−18, −6.9)

PD = Proportion difference; CI = Confidence interval.

Positive (Yes) responses from students were considered

*Weighted percentage for rural/urban balance and school size.

^†^Multiple answers under this variable.

#### Changes in school absenteeism

The statistics show a decrease in girls' absenteeism during menstruation in all schools overall. Menstruation-related absences among girls decreased from 25% in 2014 to 14% in 2018 (PD: −11; CI: −6.1 to −16). Absenteeism reduction was more predominant in urban schools than in rural schools (PD 18 vs PD 10). Similarly, the average number of days missed declined from 2.8 in 2014 to 2.5 in 2018 (PD: −0.33; CI: −0.57 to −0.10). The proportion of girls who missed classes during menstruation also reduced significantly from 40% to 30% (PD: −11; CI: −17, −4.9).

#### Reasons for missing schools during menstruation

The reasons for missing school during menstruation were investigated, and some significant changes were identified over time. The number of students reporting a lack of a separate place to change rags/clothes ascended from 8% in 2014 to 14% in 2018 (APD: 5.7; CI: 1.1–10). The percentage of parents who forbade their children from leaving the house when they were menstruating dropped from 26% in 2014 to 14% in 2018 (APD: −13; CI: −18 to −6.9).

### School absenteeism and associated factors

We performed a chi-squared test to see the association among school absenteeism and other factors. The variable time changes showed a significant relationship with absenteeism (*p* < 0.01). Adolescent schoolgirls in 2018 (14%) showed a lower rate of absenteeism compared to 2014 (25%). A similar association was found, where absenteeism was lower for those students who believed menstruation did not interfere with school performance (*p* < 0.01). School type and students’ education level were significantly associated with absenteeism (*p* < 0.05). Among school facilities, adolescents receiving any info regarding MHM from school (*p* < 0.001) and separate place for changing menstrual materials were associated with reduced absenteeism (*p* < 0.05) (Table 6).

**Table 6.** School absenteeism and associated factors.

Variables	Absenteeism		
	Yes	No	*P* value
Time			
2013	25%	75%	0.003*
2018	14%	86%	
Age			
9–12	26%	74%	0.031*
13–17	16%	84%	
Area			
Rural	21%	79%	0.446
Urban	16%	84%	
School type			
Primary school	25%	75%	0.019*
Secondary school	10%	90%	
Education level of students			
Primary	28%	72%	0.010*
Secondary	12%	88%	
Knowledge of menstruation before menarche			
Yes	18%	82%	0.175
No	23%	77%	
Believe menstruation interferes with school performance			
Yes	35%	65%	0.002*
No	12%	88%	
Received any information regarding MHM from school			
Yes	9%	91%	<0.001*
No	22%	78%	
Parent’s restriction in going out during menstruation			
Yes	29%	71%	0.197
No	19%	81%	
Using old clothes			
Yes	23%	77%	0.246
No	17%	83%	
Separate place for changing MHM materials			
Yes	12%	88%	0.012*
No	21%	79%	
Separate latrine and urine facility			
Yes	10%	90%	0.05*
No	22%	78%	

*(Statistically significant <=0.05).

### Association between school absenteeism and associated factors

The findings of a multivariate GEE model that examined the factors related to adolescent girls missing school as a result of menstruation showed that changes in time had a significant negative association with absenteeism in the unadjusted analysis, with a coefficient of −11 (CI: −16, −6.1, *p* < 0.001). The association was still significant after controlling for other factors, with a value of −10.29 (CI: −15.81, −4.77, *p* < 0.001) (Table 7).

**Table 7.** Factors associated with school absenteeism among adolescents.

Variable	Unadjusted		Adjusted	
Outcome variable Absenteeism	Coefficient (CI)	*P* value	Coefficient (CI)	*P* value
**Exposure variable**				
Changes in time	**−11 (−16, −6.1)**	<.001*	**−10.29 (−15.81–4.77)**	<0.001*
**Independent variables**				
Age			−2.41 (−5.17–0.34)	0.086
Area			**−5.6 (−10.06–1.14)**	0.014*
School type			−4.59 (−9.64–0.46)	0.075
Education level of students			**−9.48 (−14.17–4.79)**	<0.001*
Knowledge of menstruation before menarche			−1.26 (−4.39–1.86)	0.427
Believe menstruation interferes with school performance			**23.32 (19.71–26.93)**	<0.001*
Received any information regarding MHM from school			0 (−0.02–0.02)	0.951
Parent’s restriction in going out during menstruation			**4.47 (0.75–8.2)**	0.019*
Using old clothes			**−4.2 (−7.6–0.79)**	0.016*
Separate place for changing MHM materials			−0.21 (−7.25–6.82)	0.953
Separate latrine and urine facility			−5.44 (−11.46–0.59)	0.077

*(Statistically significant <=0.05).

Several independent variables were found to be significantly associated with higher rate of school absenteeism of adolescent girls. A higher likelihood of absenteeism was associated with girls living in rural areas (area), with a coefficient of −5.6 (CI: −10.06, −1.14, *p* < 0.01). Similar to this, lower educational levels of students were associated with increased absenteeism, with values of −9.48 (CI: −14.17, −4.79, *p* < 0.001). With a coefficient of −2.41 (CI: −5.17, 0.34, *p* > 0.05), age exhibited a non-significant association (Table 7).

Girls who believed that menstruation interferes with academic performance showed higher percentage of absenteeism with a coefficient of 23.32 (CI: 19.71, 26.93). Parental restrictions against leaving the house while having menstruation were also associated with increased absenteeism, with a coefficient of 4.47 (CI: 0.75, 8.2, *p* < 0.01). Girls who wore old clothes to manage menstruation were associated with higher rates of absenteeism from school (coef. −4.2, CI: −7.6, −0.79) (Table 7).

Additional variables, such as comprehending menstruation prior to menarche, receiving MHM information from the school, having a separate location for changing MHM materials, and having a separate latrine and urine facility, did not demonstrate any conclusive association with reduced absenteeism.

## Discussion

The study identified a number of factors that were associated with adolescent girls’ menstrual-related absences from school. The findings revealed that absenteeism rates significantly declined between 2014 and 2018. The GEE model indicated that increased absenteeism rates were linked to elements including rural residence, poorer educational attainment of students, parental restriction on leaving the house during menstruation, believing that menstruation affects school performance, and the use of old clothing for MHM.

The outcomes of our study show a significant trend in the context of menstrual-related school absenteeism. From 2014 to 2018, a five-year period, we observed a significant reduction in the proportion of adolescent girls missing school because of their menstruation. This decline is reflected in the average number of missed school days, which decreased from 2.8 to 2.5. The findings of our study are consistent with the evolving nature of school attendance since both urban and rural students’ absence from school decreased from 25% to 14% throughout this time. Remarkably, these findings reflect the findings of an article, how MHM programme is contributing to reduced absenteeism over time [32]. Notably, our findings are similar to previous studies undertaken in South Africa and Ghana [33,34]. However, our findings are in contrast to research undertaken in Ethiopia and Uganda [33,35–37] and illustrate that the prevalence of such absenteeism can vary considerably between different geographical regions and contexts.

In terms of area of living, our study identified that girls in rural areas are more likely to miss school due to menstruation than their urban counterparts. This finding is identical to a study conducted in Indonesia, which revealed that living in a rural area was substantially associated with school absenteeism. According to the findings of that study, girls in rural regions were roughly four times more likely than their urban counterparts to be absent from school due to menstruation [38]. Furthermore, comparable results were revealed by two Indian studies carried out in West Bengal and Pune. In the Pune study, 76.2% of rural girls reported school absenteeism due to menstruation, whereas 39% of adolescent girls in a rural secondary school reported absenteeism for the same reason in the West Bengal study [39,40]. This similarity supports the idea that the issues associated with menstrual-related absenteeism can be increased in rural settings, necessitating tailored interventions to address this issue.

Additionally, our study indicated a substantial positive association between higher absenteeism and the belief that menstruation adversely affects academic performance. This result corresponds with studies from Delhi, India, where 11.9% of girls reported missing examinations because of their menstruation [41]. In the study by Ahmed and Piro, a significant 62% of students admitted that their menstruation had affected their grades or caused them to miss exams, while 57% acknowledged its effect on participation in class and presentations [42]. Similar findings were seen in another study, which showed that more than half of respondents (57.8%) thought menstruation had adversely affected their academic performance or ranking since menarche [42].

Furthermore, our study found a correlation between parental restrictions preventing girls from leaving the house when on their menstruation and higher absenteeism rates, as shown by a coefficient of 4.47 (CI: 0.75, 8.2, *p* < 0.01). This finding, which is consistent with those of earlier research conducted in various contexts, highlights the important influence that cultural and social standards have on girls’ school attendance [41,43].

Besides, our study identified a negative coefficient of −4.2 (CI: −7.6, −0.79, *p* < 0.01), indicating a significant association between higher absenteeism from school and the use of old clothing to manage menstruation. This supports the idea that insufficient menstruation management practices may be linked to higher absence rates, reinforcing the significance of promoting thorough menstrual hygiene practices to encourage regular attendance and academic engagement. This finding is also consistent with other prior research that found similar associations [44,45].

The impact of educational policy reforms in Bangladesh between 2014 and 2018, such as legislating gender-separate facilities and employing female teachers for MHM education, reflects a major movement towards a conducive learning environment. The incorporation of MHM into the National Curriculum has generated a pleasant, inclusive learning environment, lowering barriers for schoolgirls and improving their educational experience [28,29]. These measures not only minimise absenteeism but also demonstrate social acceptance of menstruation hygiene’s critical role in ensuring equal educational opportunities.

It is necessary to ensure that girls receive appropriate information in order to properly handle menstruation-related problems [46]. There is a growing need to incorporate MHM into integrated WASH programmes, because girls require basic sanitation to maintain safe menstrual hygiene at their schools with proper facilities [10]. Girls’ attitudes towards menstruation can be changed through education sessions. Several studies found that providing girls with practical examples of MHM as well as improved WASH facilities was more effective [47,48]. Our findings highlight the value of targeted approaches for addressing MHM and school absenteeism. For better menstrual health outcomes and to lower absenteeism, comprehensive school-based programmes that include MHM information, access to hygiene materials, and supportive environments are essential.

## Strength and limitation

The analysis on school absenteeism is strengthened by the use of nationally representative datasets, the Bangladesh National Hygiene Baseline Survey 2014 and National Hygiene Survey 2018. They have a longitudinal design that enables analysing changes over time and a high sample size that improves statistical power. The reliability of standard data collection is ensured, and thorough analysis is made possible by the availability of appropriate variables. However, limitations exist, such as the inability to evaluate variables like parental education and employment status and family income levels. The study depends on participant self-reported data, which may be susceptible to recall bias and social desirability bias. Participants may give responses that they believe are socially acceptable, which may lead to overestimation or underestimation of particular behaviours. Furthermore, the study’s dependence on cross-sectional data makes it difficult to establish causal relationships.

## Conclusion

The study identifies a mixed pattern of MHM awareness and gaps. The effects of insufficient MHM are less understood, despite an increase in knowledge of menstruation before menarche. In order to address this, it is crucial that school curricula be reviewed, with a focus on the consequences of improper MHM practices and appropriate menstrual product disposal methods. Given the sensitivity of MHM, it is essential to provide school-teachers comprehensive training and ongoing capacity building. This enables them to sensitively and effectively deliver MHM education. Furthermore, considering the pivotal role of district educational authorities in delivering MHM education, consistent monitoring and evaluation of teachers’ performance in imparting MHM education are critical. This not only ensures that the curriculum’s objectives are met, but it also ensures that students receive accurate and supportive MHM instruction. Additionally, the government is urged to address and eliminate the significant sanitary facilities imbalance between rural and urban areas. Even though the use of sanitary pads has increased noticeably, poor disposal practices continue, highlighting the need for research and funding into proper disposal techniques and environmentally suitable alternatives. The updated curricula should incorporate this knowledge. Notably, the study finds a relationship between parental restrictions and absenteeism rates in addition to a substantial positive association between increased absenteeism and the perception that menstruation has an impact on academic performance. In order to effortlessly incorporate MHM into larger WASH programmes, recommendations include developing comprehensive school-based MHM programmes that provide education, access to hygiene materials, and supportive environments. Government-led initiatives are supplemented by collaborative efforts involving NGOs, private actors, and other stakeholders. This supports the findings of the study and encourages girls to attend healthier academic environments and manage their menstrual hygiene effectively.

## Data Availability

The data described in this study are accessible upon request from the corresponding author. Due to privacy and ethical restrictions, the data are not publicly accessible.

## Paper context

This study highlights a positive shift in Bangladesh, where menstrual-related school absenteeism among adolescent girls significantly declined from 2014 to 2018. Moreover, the study found that absenteeism is influenced by living in rural areas, parental restrictions, lower student education levels, belief in menstruation’s effects on academics, and using old cloth. These findings support education and MHM strategies to reduce absenteeism and improve girls’ education in Bangladesh and beyond.
